# The Chemokine System in Oncogenic Pathways Driven by Viruses: Perspectives for Cancer Immunotherapy

**DOI:** 10.3390/cancers14030848

**Published:** 2022-02-08

**Authors:** Géraldine Schlecht-Louf, Claire Deback, Françoise Bachelerie

**Affiliations:** Microbiome and Immunosurveillance, Université Paris-Saclay, INSERM, Inflammation, 92140 Paris, France; geraldine.schlecht-louf@universite-paris-saclay.fr (G.S.-L.); claire.deback@universite-paris-saclay.fr (C.D.)

**Keywords:** chemokine, oncogenic viruses, immunotherapy, cancer

## Abstract

**Simple Summary:**

Oncoviruses are viruses with oncogenic potential, responsible for almost 20% of human cancers worldwide. They are from various families, some of which belong to the microbial communities that inhabit several sites in the body of healthy humans. As a result, they most often establish latent infections controlled by the arsenal of human host responses that include the chemokine system playing key roles at the interface between tissue homeostasis and immune surveillance. Yet, chemokines and their receptors also contribute to oncogenic processes as they are targeted by the virus-induced deregulations of host responses and/or directly encoded by viruses. Thus, the chemokine system offers a strong rationale for therapeutic options, some few already approved or in trials, and future ones that we are discussing in view of the pharmacological approaches targeting the different functions of chemokines operating in both cancer cells and the tumor microenvironment.

**Abstract:**

Chemokines interact with glycosaminoglycans of the extracellular matrix and activate heptahelical cellular receptors that mainly consist of G Protein-Coupled Receptors and a few atypical receptors also with decoy activity. They are well-described targets of oncogenic pathways and key players in cancer development, invasiveness, and metastasis acting both at the level of cancer cells and cells of the tumor microenvironment. Hence, they can regulate cancer cell proliferation and survival and promote immune or endothelial cell migration into the tumor microenvironment. Additionally, oncogenic viruses display the potential of jeopardizing the chemokine system by encoding mimics of chemokines and receptors as well as several products such as oncogenic proteins or microRNAs that deregulate their human host transcriptome. Conversely, the chemokine system participates in the host responses that control the virus life cycle, knowing that most oncoviruses establish asymptomatic latent infections. Therefore, the deregulated expression and function of chemokines and receptors as a consequence of acquired or inherited mutations could bias oncovirus infection toward pro-oncogenic pathways. We here review these different processes and discuss the anticancer therapeutic potential of targeting chemokine availability or receptor activation, from signaling to decoy-associated functions, in combination with immunotherapies.

## 1. Preamble

Several groups of human viruses have been described to cause about 15–20% of the global human cancer burden [1,2,3]. These tumor viruses or oncoviruses include, so far, the RNA human T-lymphotropic virus type I (HTLV-1) and hepatitis C virus (HCV) and DNA herpesviruses (HHVs) such as the Epstein–Barr virus (EBV or HHV-4) and Kaposi’s sarcoma-associated virus (KSHV or HHV-8), the hepatitis B virus (HBV), Merkel cell polyomavirus (MCPyV), and several members of the human papillomaviruses family (HPV) [4]. The potential oncogenic role of Human cytomegalovirus (HCMV) in several cancers has been recently reviewed [5]. Human herpesvirus-6 and adeno-associated virus-2, which might also emerge as tumor viruses and human immunodeficiency virus (HIV-1), indirectly associated with cancer risk via the immunosuppression it induces [6], will not be discussed in this review.

Manifold evidence over the past decade has emphasized that some oncoviruses (HHVs; polyomaviruses, HPVs) belong to virus communities found in asymptomatic, healthy individuals that have coevolved with their hosts [7]. This important literature stresses that oncoviruses are not obligatory pathogens and that their persistence in several human body sites (e.g., skin, blood, lung, gastrointestinal tract), which manifests as latent or chronic infection (i.e., the undetectable production of the virus versus a continued one), might go beyond commensalism and contribute to human health [8]. Besides, resident viruses (i.e., the virome) might actively interact with the other microorganisms composing the microbiome that colonize healthy humans [8]. Thus, oncovirus infections do not lead to cancer as long as they are controlled by their human host and their oncogenic potential is not triggered by additional risk factors from their environment or host. While these complex host-oncovirus relationships remain largely unexplored, there are some clues regarding the host susceptibility factors which, together with viral oncoproteins, participate in the shift toward pathogenesis and tumorigenesis.

Despite structural and genomic differences, oncoviruses convey the hallmarks of cancer on the host cell through convergent mechanisms, as exemplified by host interactome and transcriptome network modifications caused by oncoviruses proteins [9,10]. The chemokine (chemotactic cytokine) system is among the pathways that are targeted by all oncoviruses (Table 1). Here, we intend first to review the current knowledge of the importance of chemokines and their receptors in oncovirus-driven tumorigenesis and introduce the mechanisms underlying their action. After introducing the chemokine system, we will focus on the role of host chemokines in the inflammation associated with oncovirus-induced solid tumors, then present the pathogenic role of herpes-encoded chemokines and chemokine receptors, and finally describe how the dysregulation of the chemokine system can play an oncomodulatory role and support the pathogenic program of oncoviruses. Lastly, we provide an overview of current and future therapeutic strategies targeting the chemokine system.

## 2. Oncoviruses’ Interplay with the Chemokine System

Chemokines and their seven-transmembrane receptors regulate organ development and play key roles in adult physiology at the interface between tissue homeostasis and immune surveillance. Beyond orchestrating directional cell migration (i.e., chemotaxis), they cover a large spectrum of cellular functions from proliferation to survival and regeneration. The interaction of most chemokines with the glycosaminoglycans of cell surfaces and the extracellular matrix help fine-tune these functions [11,12]. Receptors are primarily G Protein-Coupled Receptors (GPCRs), which, in response to chemokines (52, so far) and several other natural ligands, induce intracellular signaling processes mediated by G proteins and arrestins. A subfamily also includes four atypical chemokine receptors (ACKRs) that share the characteristics of not signaling through G proteins but arrestins and having the capacity to promote chemokine internalization and degradation [13].

By doing so, ACKRs control chemokine abundance and distribution in tissues and in circulation and thus indirectly regulate the function of conventional GPCRs [14]. ACKRs can thus impact cancer progression by shaping chemokine availability within the tumors, thereby decreasing GPCR-mediated tumor cell metastasis and immune cell infiltration. Moreover, they can directly contribute to tumorigenesis. This dual role is illustrated for ACKR3 [15], which shares the CXCL12 chemokine with CXCR4 [16] and modulates CXCL12/CXCR4 responses [17]. It manifests by ACKR3 upregulation in several types of cancer, including virus-induced cancers [18], in both the primary tumor cells and tumor-associated endothelial cells, also substantiating the importance of ACKR3 activation by its repertoire of natural chemokine (CXCL12/CXCL11) and non-chemokine ligands (including macrophage migration inhibitory factor (MIF), adrenomedullin, and opioids) [19,20,21]. In contrast, other atypical non-G protein signaling chemokine receptors such as CC chemokine-receptor-like 2 (CCRL2), devoid of scavenging properties, can favor anti-tumor responses. This can be accomplished by a transpresentation of the chemotactic non-chemokine chemerin by CCRL2 to CMKLR1, heterodimerization with CXCR2 [22], or interaction with Toll-Like Receptor 4 (TLR4), which facilitates CCRL2 retainment in the cell surface of tumor-associated macrophages (TAMs) and potentiates further anti-tumor CD8+ T-cell responses [23].

**Figure 1:**
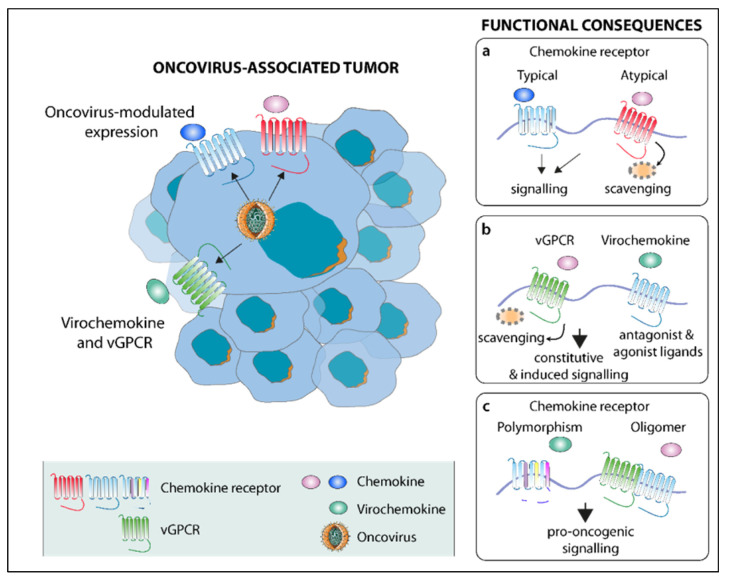
Interplay between oncoviruses and the chemokine system. By modulating the expression of chemokines and their receptors and encoding for viral homologs (left part), oncoviruses can hamper the functioning of chemokines and their typical and atypical human receptors through (**a**) increased activation or aberrant signaling; (**b**) virochemokines (e.g., vCCL-1-3) and vGPCRs (e.g., ORF74) acting as ligands and receptors of their human homologs, respectively; and (**c**) GOF mutants of human receptors (e.g., CXCR4) or potential oligomers formed between vGPCRs and human receptors (e.g., BILF1/CXCR4).

Along with the proper functioning of the chemokine system to ensure the timely control of immune responses, chronic inflammation, which is a hallmark of cancer, often evolves in a context of the abnormal and uncontrolled expression and/or function of the chemokines and/or their receptors in the tumor microenvironment [24]. Such dysfunctions, by a succession of interactive and self-reinforcing feedback loops actioned through the receptor, chemokine, and viral proteins, further foster all stages of tumor progression from cancer cell proliferation, survival, and migration to tumor angiogenesis, invasion, and metastasis. Moreover, some oncoviruses, namely KSHV and EBV, encode viral homologs of human chemokines or chemokine receptors (virochemokines and viral G protein-coupled receptor or vGPCRs (Table 1)) that can contribute to tumorigenesis via the constitutive activation of paracrine angio-proliferative and pro-inflammatory pathways [25]. Chemokines and their receptors can also be targets of oncogenic viral proteins translating into the deregulated expression or epigenetic modification of their genes. Conversely, some deregulated chemokine signaling pathways were described as potent activators of oncovirus-driven tumorigenesis at the cost of the replication of these commensal viruses. In this chapter, we will review the mechanistic links underlying the interplay between oncogenic viruses, chemokines, and their receptors (Table 1 and Figure 1).

### 2.1. From Chronic Inflammation to Oncogenic Transformation: Focus on Hepatitis B and C Viruses’ Interplay with the Chemokine System

Diseases driven by chronic infection with HBV and/or HCV progress at a variable rate, from active inflammation to fibrosis and possibly cirrhosis, the onset of which precedes the development of hepatocellular carcinoma (HCC) [26]. According to the World Health Organization, in 2019, 0.82 and 0.29 million individuals died of HBV- and HCV-related HCC, respectively, which represent 96% of the deaths caused by viral hepatitis [27]. On the one hand, the chemokine system participates in the resolution of acute HCV infection via strong T-cell responses that are promoted along with the upregulation of chemokine expression (e.g., CXCR3 and CCR5 ligands) in HCV-infected liver cells [28,29]. While the role of chemokines in the clearance of acute HBV infection is less established [29], the timely mobilization of relevant immune cells and the increased expression of chemokines (e.g., primarily CXCR3 ligands) were also reported in HBV-positive hepatocytes and sera from infected patients [30]. On the other hand, the detrimental roles of chemokines and their receptors in the development and maintenance of chronic and uncontrolled inflammation that fosters fibrosis, cirrhosis, and HCC development are well known [31,32]. Several chemokines and receptors belong to the panel of genes whose expression levels in the liver of HCV-associated early-stage cirrhosis patients are significantly associated with the risk of progression [32]. As such, the upregulated expression of CXCL6, as a possible consequence of that of the bromodomain-containing protein 4, was proposed to participate in the development and progression of HBV-induced liver fibrosis [33]. Moreover, the upregulation of the CXCL10/CXCR3 chemokine/chemokine receptor pair has been correlated with the progression toward liver disease [34]. The attractive possibility that chemerin levels might be decreased in the HCC of viral etiology is, however, debated [35]. Beyond epigenetic mechanisms that might account for the upregulation of chemokine and receptor expression by HBV- and HCV-derived products [29], several chemokine genetic polymorphisms that positively correlate with oncoviral-induced liver diseases were recently reported. Hence, polymorphisms in *CCL2* or *CXCL9-11* were found in patients developing HCV-related fibrosis [29]. Selective polymorphisms of *CXCL10* were also identified in patients with chronic HBV infection and suspected to be susceptibility factors in the pathogenesis of HBV infection [36,37]. Such dysfunctions of the chemokine system can drive the uncontrolled recruitment of immune cells in the liver, such as that of CXCR3-expressing natural killer (NK) cells and activated T cells and B cells by the way of hepatocyte-secreted CXCL10 [34]. They also can jeopardize effective immune responses through the recruitment of regulatory T cells (Tregs) in the liver of patients chronically infected by HCV, which foreshadows the immunosuppressive HCC tumor microenvironment (TME) [38]. Thus, targeting chemokines and their receptors could thus be a relevant therapeutic strategy, in combination with the immune checkpoint inhibitors currently being studied or in clinical trials aimed to treat HCC.

### 2.2. Oncovirus-Encoded Products’ Relationship with the Chemokine System

#### 2.2.1. Viral Chemokines and GPCRs: Focus on Epstein-Barr Virus and Kaposi’s Sarcoma Associated Virus

The eight HHVs have large genomes encoding more than a hundred open reading frames (ORFs). Hence, beta-herpesviruses (HCMV, HHV-6, and HHV-7) and the two oncogenic gamma-herpesviruses (EBV and KSHV) encode viral chemokines or “virochemokines” excreted by the infected cells and chemokine receptor-like proteins homologous to the human GPCRs (vGPCR) [5]. Here we will review the reported mechanisms by which these viral mimics may contribute to oncogenesis.

- Epstein-Barr virus (EBV)

Besides causing mononucleosis, EBV (HHV-4) is responsible for lymphoproliferative diseases and solid tumors derived from epithelial cells such as nasopharyngeal and gastric carcinoma, and, more rarely, from mesenchymal cells such as leiomyosarcoma (Table 1). While immunosuppression is one risk factor for the development of EBV-induced malignancies, such as post-transplant lymphoproliferative diseases and acquired immunodeficiency syndrome (AIDS)-lymphoma, other cancers can occur without immunosuppression (Burkitt’s lymphoma, Hodgkin’s, and non-Hodgkin’s lymphoma), and the oncogenic mechanisms remain still relatively unknown. To date, one vGPCR or BILF1 (for small BamHI fragment I) has been identified in the EBV genome. BILF1 displays some sequence similarity with CXCR4 (15%), but no ligand of BILF1 has been reported to date. It signals constitutively by coupling to the Gαi class of G proteins [39], thus inhibiting adenylyl cyclase and constitutively modulating various cellular signaling pathways, depending on the cell context [40]. An additional signaling effect of BILF1 lies in its capability to hijack the signal transduction pathway from neighboring human host GPCRs via Gαi scavenging or the inhibition of chemokine binding (e.g., CXCR4) [41] or heterodimer formation (e.g., CCR9, CCR10, CXCR3, and CXCR4) [42]. These effects may account for the suspected immunomodulatory roles of BILF1 together with its reported capacity to downregulate MHC class I molecules, mainly at the late phase of the virus lytic cycle, leading to the impairment of T cell recognition [43,44]. Similar properties are endowed by the vGPCRs US28 [45] and ORF74 [46], encoded by HCMV and KSHV, respectively, and also suggested to have an important role in the latent viral life cycle [5]. While BILF1 is an early-produced protein, apparently not required for lytic replication [47], its reported expression in EBV-transformed B cell lines and EBV-induced tissue cancer [48,49] may be indicative of its role in immune evasion by EBV-tumor cells [41,50].

- Kaposi’s sarcoma-associated virus (KSHV)

KSHV can cause the development of three malignant proliferative disorders, namely Kaposi’s sarcoma (KS), the primary effusion lymphoma (PEL), and the Multicentric Castleman’s disease (MCD) [51]. More often aggressive in immunosuppressed patients, they complicate AIDS and organ transplantation. KS is a multifocal, highly vascularized neoplasm, composed of KSHV-infected spindle-shaped cells of endothelial origin, extensive angiogenesis, and a cellular infiltrate composed of macrophages, neutrophils, mast cells, and lymphoid cells. PEL is a rare, rapidly fatal B-lymphoma associated with KSHV or KSHV/EBV infection, which presents in AIDS patients as a pleural or pericardial effusion and, more rarely, as a solid mass in the lymph nodes, lungs, or the gastrointestinal tract [52]. MCD is a lymphoproliferative disorder characterized by large plasmablastic cells and vascular proliferation that can progress into lymphoma [53]. The expression of the viral-encoded GPCR and chemokines might directly contribute to the angioproliferation induced by KSHV [54]. ORF74 vGPCR expression, controlled by the major latent LANA viral protein, is detected in a fraction of KS lesions and PEL cell lines [55]. ORF74 displays the highest (27%) sequence similarity with CXCR2. It binds to chemokines from the CXC family (CXCL1-3, CXCL5-8) as well as CXCL4, CXCL10, CXCL12, CCL1, CCL5, and the virochemokine vCCL2 encoded by KSHV [56,57], and also displays the ability to signal in a constitutively active, ligand-independent manner. Beyond the critical requirement of ORF74 for efficient viral replication [55], much evidence also highly suggests its pro-oncogenic contribution to KSHV-induced oncogenesis [58,59,60] through the ligand-dependent and -independent signaling pathways involved in enhanced inflammation and immunomodulation [61]. KSHV also express additional non-structural proteins suggested to contribute to tumor progression, among which are three virochemokines, vCCL1 (encoded by *ORFK6*), vCCL2 (encoded by *ORFK4*), and vCCL3 (encoded by *ORF4.1*), as well as vIL-6, the viral homolog to human IL-6 (encoded by *ORFK2*) [62,63,64]. vCCL1 and vCCL3 bind to a restricted panel of GPCRs (i.e., CCR5/CCR8 and CCR4/XCR1, respectively) while vCCL2 interacts with a broader range of CC GPCRs (CCR1, CCR2, CCR3, CCR5, CCR8, and CCR10) as well as with XCR1, CXCR4, and CX3CR1. To our knowledge, it has not been reported whether the three virochemokines and, more generally, the pro-oncogenic viral genes, are differentially expressed in the three KSHV-related diseases. As reviewed elsewhere [64], the interactions between virochemokines and human GPCRs can promote a Th2 response in the context of KSHV replication, whereas they activate anti-apoptotic and angiogenic pathways that might contribute to KSHV pathogenesis. Moreover, ACKRs may regulate the availability of the virochemokines for human GPCRs and their role in the KSHV life cycle in view of the recently reported capacity of ACKR3 to act as a scavenger for vCCL2 [65].

#### 2.2.2. Oncovirus-Encoded microRNAs

Among the epigenetic processes used by viruses to remodel the host transcriptome, microRNA (miRNA) encoded by viruses or cellular miRNA modified by viruses are suggested mechanisms whereby chemokines and their receptors are targeted by oncoviruses. MicroRNAs (miRNAs) are small non-coding RNAs with a length of 21–23 nucleotides generated from both protein-coding and non-coding transcripts. To date, several oncoviruses have been found to encode miRNAs differently expressed during their life cycle. Among them, EBV-miRNAs, KSHV-miRNAs, and MCPyV-miRNAs directly subvert the host defense system, notably by regulating immune-related gene expression, including chemokines and their receptors.

- Epstein–Barr virus (EBV)

EBV encodes a total of 44 miRNAs derived from two genomic regions: the BAMHI-A region rightward transcript (BART) and the BamHI fragment H rightward open reading frame 1 (BHRF1) clusters [66]. EBV miRNAs are expressed during the viral lytic cycle and also modulate the expression of numerous viral (i.e., the latent membrane protein LMP2A and polymerase BALF5) and cellular targets during all forms of viral latency [67]. BART-miRNAs can be detected in Burkitt’s lymphoma cells, NK/T-cell lymphoma cells, and epithelial tumor cells such as nasopharyngeal carcinoma and gastric carcinoma cells. In contrast, BHRF1-miRNAs are solely detected in EBV-B-cell tumors. With regard to the interference with chemokine signaling, miR-BHRF1-3 downregulates the expression of CXCL11, a CXCR3 ligand, as observed in AIDS-related diffuse large B-cell lymphomas [68]. Another strategy of EBV-driven immunomodulation is the capacity of the virus to induce the expression of cellular miRNAs that, in turn, may reinforce viral latency. Hence, miR-146a, which is highly up-regulated in EBV-associated tumor cells, is reported to down-regulate the expression of CXCR4, a process that may participate, together with viral proteins (e.g., latent membrane protein-1 LMP1), in the immune evasion of EBV-tumor cells [69].

- Kaposi’s sarcoma-associated virus (KSHV)

During its latent phase, KSHV expresses a genomic region encompassing 25 miRNAs in addition to genes encoding for FLIP (vFLIP), v-cyclin, kaposin, and LANA (the latency-associated nuclear antigen). Constitutively expressed during latency, KSHV-encoded miRNAs are reported to participate in the establishment of viral latency and also in the development of KSHV-associated diseases by modulating cellular pathways that would favor the angiogenesis and dissemination of the tumor cells and by interfering with host immune surveillance. Among them, miR-K10a downregulates the expression of the CCL2 chemokine ligand of CCR2 and participates with other miRNAs in jeopardizing cellular responses, notably mediated by tumor necrosis factor-related weak inducer of apoptosis (TWEAK), type I interferon (IFN), or the IL-1 receptor (IL-1R) (e.g., miR-K11 and miR-K7, miR-K9, and miR-5) [70,71].

- Merkel cell polyomavirus (MCPyV)

Merkel cell carcinoma (MCC) is a rare, aggressive, poorly differentiated neuroendocrine cutaneous carcinoma associated with Merkel cell polyomavirus (MCPyV) clonally integrated within malignant cells, most often in aging and chronically UV-damaged skin [72]. In primary MCC tumors, the presence of CD8+ T cells, associated with the upregulation of CCL19 and CCR2 expression, is a significant predictor of good outcomes [73]. However, in most MCC tumors, only a limited number of CD8+ cells penetrate the malignant tumor mass that rather tends to form peripheral clusters together with inflammatory cells, suggesting the setting of abnormal signaling as a consequence of the deregulated expression of numerous chemokines and receptors (e.g., CXCL9-12, CXCR4, and CXCR3) [74]. Whether MCPyV-latent infection participates in this deregulation remains to be determined. MCPyV has been reported to encode a pre-miRNA located on the antisense strand of Large T (LT) ORF, which results in two MCV-miR-M1-3p and 5p miRNAs present in ~50% of MCC tumors [75]. Beyond their role in viral persistence by limiting viral DNA replication [76], MCV-miR-M1 can target the intrinsic immune response against double-stranded DNA viruses (e.g., Speckled Protein 100) [77]. They can also interfere with host immune surveillance, as exemplified by the downregulation of the expression and secretion of CXCL8 by MCC cell lines [77], an important chemokine in the antiviral response in the skin, also targeted by EBV and KSHV [78].

#### 2.2.3. Viral Oncogenes

- Human T-lymphotropic virus type I (HTLV-1)

HTLV-1, the first discovered human oncovirus, is a retrovirus that causes adult T-cell leukemia/lymphoma (ATLL) in approximately 5% of the infected individuals after a latency period that lasts several decades. There are four subtypes of ATLL, namely acute, lymphoma, chronic, and smoldering, with different levels of aggressiveness. With cytotoxic chemotherapy, patients’ median survival is less than 1 year [79]. A characteristic feature of ATLL is organ infiltration, which is governed by chemokine receptor expression by transformed cells. Hence, ATLL cells can be found in the lymph nodes, spleen, liver, skin, and gut [80]. Two viral proteins, Tax and HTLV-1 basic leucine zipper factor (HBZ), have been reported to play a crucial role in the transformation of HTLV-1-infected CD4^+^ T cells and the development of ATLL. The transcriptional activator and oncoprotein Tax, which is encoded by the pX region of the HTLV-1 genome, is responsible for the initiation of cell transformation. Tax being the most immunogenic viral protein, T cell clones that have deleted the pX region and lost Tax expression due to immunologic pressure are selected over time [81]. HBZ, which is encoded by the antisense strand, is then critical for transformed cell survival [82]. The chemokine system has been linked to ATLL pathogenesis at multiple levels [83]. Hence, ATLL cells have been reported to overexpress several chemokines and chemokine receptors, with effects on their survival, migration, and homing. Tax promotes NFκB-dependent transcriptional activity, a mechanism that accounts for the increased expression of several chemokines, including CCL1, CCL2, CCL3, CCL4, CCL5, CCL22, CXCL8, and CXCL12 by ATLL cells [84,85,86,87,88,89,90,91,92]. CCL1 was reported to create an anti-apoptotic autocrine loop upon CCR8 engagement in ATLL cells [93]. CCL3 and CCL4 chemokines were shown to promote the integrin-dependent adhesion of ATLL cells to the endothelium, thereby favoring their transmigration [86]. In addition, Tax-induced CXCR4 expression was shown to promote ATLL cell migration toward CXCL12-rich environments [87], a process that was proposed to contribute to ATLL cell homing in the skin [94]. Tax was further reported to modify G-protein β subunit subcellular localization, thus promoting CXCL12/CXCR4-mediated signaling [95]. ACKR3 expression was also reported to be induced by Tax in T-cell lines, albeit its expression by ATLL cells was highly heterogeneous, with a proposed role in cancer cell survival [96]. A high level of CCR4 expression is a hallmark of most ATLL cells [97,98], the leukemic cells expressing this receptor in 90% of ATLL cases [99]. CCR4 overexpression, induced by HBZ [91] was reported to increase the proliferation of ATLL cells and their migration towards CCL17 and CCL22, thus contributing to skin infiltration [92,99,100]. In addition, ATLL cells also produce CCL17 and CCL22 [101]. The expression of CCL22, induced by Tax [92], has been proposed to favor the recruitment of CCR4^+^ T cells, which are mostly Th2 cells, FoxP3^+^ Tregs, and cutaneous lymphocyte antigen-positive skin-homing T cells [97], to the viral synapse, which would lead to their preferential infection. Further underlining the importance of the CCL17/CCL22/CCR4 signaling axis in ATLL pathogenesis, somatic gain of function (GOF) mutations in *CCR4*, detected in 26 to 29% of ATLL cases [102,103], result in increased cell migration toward CCL17 and CCL22, together with impaired CCR4 internalization [102]. Moreover, GOF mutations in *CCR7* have been also reported in 11% of ATLL cases [103], and in patients with lymphoid organ infiltration, ATLL cells were further shown to upregulate CCR7 whose expression in CD4^+^CCR4^+^ ATLL cells is associated with aggressive ATLL while its absence is associated with progressive indolent ATLL [80,104]. Finally, the anti-CCR4 monoclonal antibody, Mogamulizumab, displays some efficacy in ATLL, as discussed later in Section 3.1. Of note, the expression of CCR9 by ATLL cells was reported to be associated with the levels of Tax and proposed as the driver of their migration to the gut [105,106].

- Human papillomaviruses (HPVs)

HPVs are non-enveloped DNA viruses that display strict tropism for human keratinocytes of skin and mucosa stratified epithelia. Recent progress in the sensitivity of sequencing methods has allowed the discovery that the HPV family includes 442 types (among them ~200 are awaiting classification approval by the International Committee on Taxonomy of Viruses; December 2021, https://pave.niaid.nih.gov/#explore/reference_genomes/human_genomes), representing the most abundant eukaryotic viruses of the human epithelia microbiome [107,108,109]. HPV chronic carriage by the general population, manifesting as an individual signature acquired early in life and stable over time, reveals that those viruses are mostly asymptomatic commensals. This opens the possibility that beneficial interactions exist between HPVs and their human host. Yet, some mucosal HPV types can induce lesions. While most of them are cleared by the immune response, a minority (10–15%) can persist and progress over decades to neoplasia and cancer. HPVs are responsible for ~5% of human cancers, including most cervical, certain anogenital, and an increasing number of oropharyngeal cancers. They are also suspected to act in synergy with UV to initiate non-melanoma skin cancers [6]. Prophylactic vaccines were an important breakthrough in preventing infection with the HPV types causing cancers [110]. Nevertheless, due to insufficient vaccine coverage and a lack of treatments clearing established HPV-associated lesions, HPV-associated cancers remain an important global health issue [111]. Thus, in the view of HPVs as contextual pathobionts [112], a better understanding of the human host and environmental factors that can bias commensal HPVs towards pathobionts would provide therapeutic paths. While these are under-studied questions due to the lack of in vivo models for the productive HPV life cycle and in vitro system cultures for cutaneous HPVs, the deregulated expression of viral oncogenes (E6, E7, and E5) associated with persistent infection has a demonstrated role in carcinogenesis. Interactome and functional analyses have shown that beyond the inactivation of tumor suppressor pathways [113], viral oncogenes have the propensity to alter a wide range of cellular protein activities. This includes chemokines and their receptors, the deregulation of which may contribute to forming a microenvironment supporting viral persistence and eventually neoplasia. As such, the downregulation of CXCL14 and CCL20 by HPV was respectively shown to jeopardize CD8+ T cell antitumor responses in HPV-tumors injected in mice [114] and associated with a reduced number of Langerhans cells in the HPV lesions of patients with the epidermodysplasia verruciformis (EV) skin disease [115]. Moreover, a mouse model of HPV-associated carcinogenesis, expressing HPV16 type oncogenes at high risk of cancer, in the basal layer of stratified squamous epithelia, has highlighted the contribution of several chemokine ligands, notably of the proangiogenic CXCR2 receptor, to the increasing inflammation of the tumor microenvironment in the course of neoplasia [116,117]. In such a model, the alteration of skin dendritic cell subsets’ migration to regional lymph nodes as a consequence of a CXCR4 GOF was dramatically associated with increased inflammation [118]. In this regard, it is noteworthy that CXCR4 GOF heterozygous dominant mutations are responsible for the rare human immunodeficiency WHIM syndrome (for Warts, Hypogammaglobulinemia, Infections, and Myelokathexis) featured by a panleukopenia and selective susceptibility to HPV-induced pathogenicity [119]. Normalizing CXCR4 function in WHIM patients upon treatment with a selective CXCR4 antagonist (AMD3100/Plerixafor) [120,121], or as a consequence of a fortuitous loss of the mutated *CXCR4* allele [122], improves the panleukopenia as well as HPV-induced lesions. These beneficial effects, also observed in a mouse model of HPV-associated carcinogenesis [123], can be accounted for by the role of CXCR4 and its chemokine ligand CXCL12 in the control of the HPV life cycle through the cooperation of mechanisms operating at the level of HPV-infected keratinocytes and their microenvironment, as well as at the global immune surveillance level. First, CXCL12 expression, absent in healthy epithelia, was observed in HPV skin or mucosal lesions [124], suggesting that HPV oncogenes can alter the expression of the chemokine and its receptors CXCR4 and ACKR3 as they do in cultured keratinocytes [125]. Then, expression of the WHIM-associated genetic variant of CXCR4 is capable of biasing the HPV life cycle toward an oncogenic program, as demonstrated in three dimensional (3D) organotypic epithelial cultures [126] in support of its role as a susceptibility co-factor for HPV-induced pathogenesis, as proposed also for genetic variants of CXCL12 [127]. Second, mice harboring WHIM-associated Cxcr4 GOF mutation models the panleukopenia reported in patients [128] in support of critical functions endowed by CXCR4 in immune-hematological processes, including homeostatic immunity, driven by a network of skin-resident immune cells [129]. In this context, proper CXCR4 signaling controls the migration and activation of skin DC subsets that appear to be critical surveillance mechanisms of the HPV life cycle [118], whose recovery in WHIM patients after blocking CXCR4 dysfunction would explain the benefit on HPV manifestations of pathogenesis [121,130]. Deciphering the pathogenesis of the inborn errors of immunity diseases marked by selective susceptibility to HPV pathogenesis [131] has highlighted the role of immune responses, of which chemokines are prominent regulators, in the control of the HPV life cycle, together with that of epithelia intrinsic immunity [111]. Thus, therapeutic options aimed at recovering or inducing cellular immunity in HPV-induced neoplastic lesions to overcome the local pro-tumorigenic inflammation are considered [132]. For instance, a recent study has provided a strong rationale for the use of checkpoint-inhibitors such as the programmed death ligand 1 (PD-L1) in the treatment of HPV-induced head and neck squamous cell carcinoma [133], where they can be combined with the chemokine receptor blockade (CXCR2, CSF1R, and CCR4) [134].

## 3. Targeting the Chemokine System as a Therapeutic Approach in Oncovirus-Associated Cancer

The identification of the specific links between the chemokine system and cancer has paved the way for targeting the different components of this system to affect cancer cell stemness, proliferation, survival, and metastasis, but also angiogenesis, as well as immune cell recruitment and imprinting in the TME. The strategies aimed at modulating the chemokine system are essentially devised as adjuvant therapies, with the objective of maximizing the beneficial effects of existing anti-cancer treatments. They become even more relevant considering the increasing number of reports unraveling the importance of tumor-infiltrating cells for the success of immune checkpoint blockade therapies. However, targeting the chemokine system can also be complex, due to the intricate relationships between the different chemokines, receptors, and atypical receptors. In this chapter, we will review the few current anti-cancer therapies, approved or in clinical trials, in cancers as well as the potential new paths for virus-associated cancers opened by recent works in the field. We will focus on the strategies selectively targeting the chemokines and their receptors, as well as the atypical receptors known to regulate prototypic GPCRs function. Considering the large number of studies in the field, we will focus on some examples of particular relevance for virus-associated cancers. Some approaches aimed at broadly modulating chemokines and their receptors, such as HDAC inhibitors that are currently being tested in HPV- and EBV-induced cancers (NCT03357757), will not be discussed in detail as immune checkpoint inhibitors currently in trials for MCC tumors [135].

### 3.1. Current Anti-Oncovirus Cancer Therapies

Only one treatment targeting the chemokine system has been approved so far in oncovirus-associated cancers. This immunotherapy is Mogamulizumab (KW-0761), a defucosylated anti-CCR4 monoclonal antibody, which was approved in 2012 for the treatment of relapsed/refractory ATLL following allogeneic hematopoietic stem cell transplantation, and in 2014 for the treatment of newly diagnosed ATLL patients, in Japan [136,137,138,139,140] (see also [141] in this issue of Cancers). The rationale for targeting the CCL17/CCL22/CCR4 chemokine axis was based on the contribution of this signaling axis to ATLL progression and tissue infiltration (see previous Section 2.2.3). The mechanism underlying Mogamulizumab efficiency is based on targeted antibody-dependent cellular cytotoxicity toward CCR4^+^ cells, which is mainly mediated by NK cells. The Mogamulizumab-induced adverse effects that have been reported include severe skin disorders or HBV reactivation and may result from Tregs depletion [142,143,144]. A complete review on Mogamulizumab can be found in this issue of Cancers [141]. The success of this pioneer therapeutic approach provides the proof of principle that targeting chemokine receptors, and, more broadly, chemokine signaling pathways, may provide efficient treatments toward virus-associated cancers.

### 3.2. Clinical Trials and Promises in Targeting Chemokines in Oncovirus-Associated Cancers

**Figure 2:**
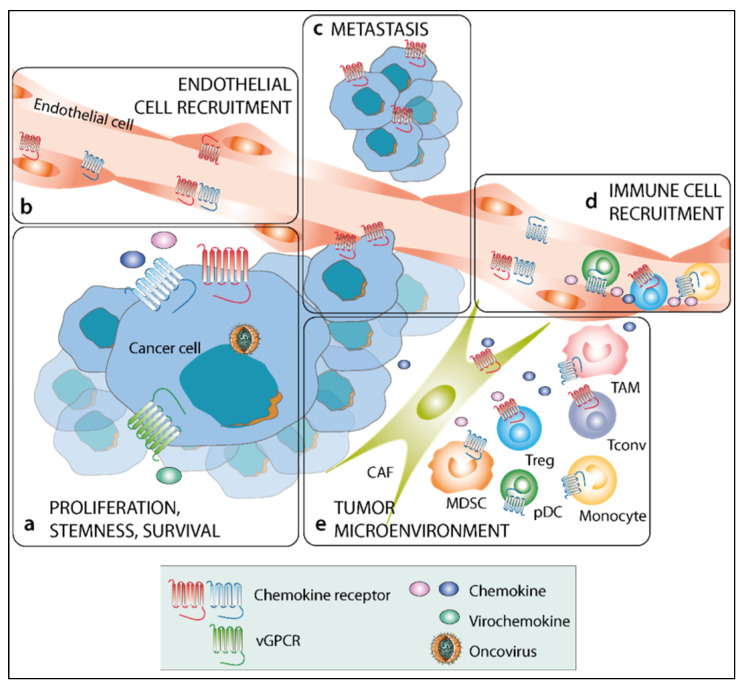
Harnessing the chemokine system to modulate oncovirus cancer features. (Viro)chemokines and their receptors can be targeted to limit (**a**) cancer cell stemness, proliferation, and survival, (**b**) angiogenesis, (**c**) metastasis, (**d**) immune cell recruitment, and (**e**) TME composition and polarization.

The deregulations in chemokine expression levels in oncovirus-associated cancers, as well as their consequences in both cancer cells and TME, provide the rationale for their targeting (Figure 2). In addition, the virochemokines produced by certain viruses are, by nature, targetable tumor-associated antigens. Thus, interfering with chemokines to adequately modulate their pro- or anti-tumoral actions appears as a relevant therapeutic option in some viral-induced cancers. Such approaches may be based on the use of high-affinity chemokine-specific ligands, including monoclonal antibodies or nanobodies (i.e., antibody fragments generated from heavy-chain antibodies from camelids [145]) to directly neutralize chemokines, but may also be achieved through the modulation of chemokine expression or the disruption of chemokine interactions with GAGs [12]. The challenge in targeting chemokines lies in their pleiotropic action, most often mediated through several receptors, which can complicate the anticipation of the intervention’s effects.

#### 3.2.1. Targeting Chemokines to Modulate Cancer Cell Functions

Chemokines can act directly on cancer cells to promote their stemness, proliferation, survival, and metastasis [146,147]. Among the chemokines reported in oncovirus-associated cancers, CCL2 and CXCL12 are known to directly support cancer cell stemness, proliferation, and survival. CXCL8 has also been reported to promote cancer cell stemness. Certain chemokines, including CCL5 and CXCL12, have further been shown to drive cancer cell metastasis. In murine mammary tumors, CCL2 secretion was also reported to indirectly support metastasis by promoting inflammatory monocyte recruitment [148]. Based on these data, a strategy aimed at neutralizing CCL2 by specific monoclonal antibodies has been tested in breast cancer models. Unexpectedly, despite the anti-metastatic action of this antibody, a pro-metastatic effect of CCL2 blockade discontinuation was reported, inviting caution when targeting CCL2 in cancer [149]. Nanobodies, known for their flexible and scalable format, their robustness, and their affinity for their targets in the nanomolar ranges, have also been devised toward CCL2, CCL5, and CXCL12 [150], thus providing promising tools for the targeted modulation of cancer cell proliferation, survival, invasiveness, and metastasis in oncovirus-associated cancers.

#### 3.2.2. Targeting Chemokines to Limit Cancer-Associated Angiogenesis

Chemokines can have indirect effects on cancer cells by promoting angiogenesis and tumor vascularization. CCL2, CXCL8, and CXCL12 were reported to promote these processes [151]. The identification of CCL2 as a pro-angiogenic factor in solid tumors led to the test of monoclonal antibodies targeting this chemokine, with the aim of counteracting its pro-angiogenic action. Along this line, the Carlumab (CNTO 888) anti-CCL2 IgG1κ mAb has been tested in various solid tumors (NCT00537368) but was discontinued due to its poor efficiency when tested in metastatic prostate cancers [152,153].

#### 3.2.3. Targeting Chemokines to Modulate Immune Cell Recruitment and/or Polarization in the TME

Chemokines are critical players in controlling the TME content in immune cells as well as immune polarization. They may be targeted in order to favor the expression chemokines that could display an anti-tumoral function in some contexts. Along this line, several studies have demonstrated that the expression of the Th1-type CXCL9 and CXCL10 chemokines can be repressed in cancer cells and their TME by epigenetic processes related to the Polycomb group proteins and the DNA methylation system (e.g., EZH2 protein and DNMT DNA methyltransferases), thereby contributing to the emergence of “cold” tumors [154,155] that resist immunotherapy. Based on these reports, the use of inhibitors targeting EZH2 or DNMT has been tested and reported to increase the production of these chemokines in the tumor bed, thereby promoting T-cell trafficking into tumors [146]. Conversely, the detrimental effects of certain chemokines, including CCL2, CCL3, CCL5, CXCL8, and CXCL12, in shaping immunosuppressive TME may also be targeted directly using chemokine-directed monoclonal antibodies or nanobodies [145,146,147] or indirectly using glycomimetics aimed at disrupting chemokines/GAGs interaction, thereby interfering with the formation of local chemokine gradients and impacting on cell trafficking [12].

### 3.3. Targeting Host Chemokine Receptors or vGPCRs in Oncovirus-Associated Cancers

The specific pattern and/or high level of chemokine receptor expression by cancer cells in oncoviral-induced cancers and the presence of vGPCRs in some cases provide targetable tumor-associated antigens and have been the basis of several anticancer strategies. The first approach consists of using the selective expression pattern of some chemokine receptors to kill cancer cells, as done for ATLL with Mogamulizumab. The second strategy consists of acting on chemokine receptors and vGPCRs signaling by using biologics and small molecules/peptides, an approach that may be used to target either cancer cells or cells from the TME.

#### 3.3.1. Targeting Chemokine Receptors and vGPCRs to Eliminate Cancer Cells

Several chemokine receptors, namely CCR4, CCR7, CCR9, and CXCR4, are substantially expressed in oncovirus-induced cancer cells. Based on the proof of principle that ATTL cells can be eliminated through CCR4, targeting other chemokine receptors has gained interest. Among these receptors, CXCR4 has been the focus of much effort, due to its involvement in many different cancer types [15]. Recently, the fusion of a nanobody specific for the second extracellular loop of CXCR4 to the Fc domain of a human IgG1 antibody was reported to mediate CCFR-CEM lymphoblastic T-cell killing through antibody-dependent cell-mediated cytotoxicity by NK cells in vitro [156]. Capitalizing on the critical contribution of the CCL17-CCR4 chemokine axis in ATLL, the use of CCL17 fused to an exotoxin of Pseudomonas was shown to kill HTLV-1-infected cells in a CCR4 and furin-dependent manner [157], and the design of T cells expressing a chimeric antigen receptor that targets CCR4 demonstrated its efficiency in lysing autologous patient-derived tumor cells [158]. The expression of vGPCRs by cancer cells is also considered a relevant target. The proof of principle of the efficiency of such an approach has been provided for US28, a constitutively activated, HCMV-encoded vGPCR mimicking CX3CR1 signaling. US28 activation is essential for virus latency in hematopoietic cells and is involved in glioblastoma and vascular diseases participating in proliferative, angiogenic, and inflammatory processes [159]. Recently, it has been shown that VUN100(vb), a monovalent nanobody targeting the extracellular domains of US28, induces HCMV immediate early antigen expression without promoting full viral reactivation, thus allowing for the HCMV-specific T-cell-mediated clearing of latently infected CD14+ monocytes [160]. This strategy offers an attractive new therapeutic approach for HCMV-positive tumors [161]. Similarly, the use of affibody molecules targeting EBV-LMP1/LMP2A proteins has been considered for treating patients with nasopharyngeal carcinogenesis [162]. In this regard, nano/intrabodies or immunotoxins targeting the vGPCR of EBV (BILF1) and KSHV (ORF74) may constitute new specific technologies against these oncovirus-induced diseases and cancers, for which there are no specific antiviral therapies to date [163].

#### 3.3.2. Targeting Chemokine Receptors Signaling in Cancer Cells

The critical contribution of chemokine receptor-mediated signaling pathways to cancer cell hallmarks has been robustly established and was further emphasized by the pro-oncogenic signaling driven by GOF mutations that have been identified for CCR4 and CCR7 in ATTL and for CXCR4 in the Waldenström macroglobulinaemia; the latter mutations being similar to those inherited causing the WHIM syndrome and promoting the oncogenic program in HPV-infected keratinocytes. These works have provided a strong impetus to identify small molecules that would antagonize or bias chemokine receptor signaling. The CXCR4 orthosteric antagonist Plerixafor was the first chemokine receptor antagonist to be approved in lymphoma and myeloma and is currently being tested in other cancers, as well as in WHIM syndrome patients to treat HPV-induced lesions [121,130]. Based on the role of CXCR4 signaling in ATLL cell maintenance and the beneficial effect of CXCR4 blockade on tumor growth reduction in vivo [164], the potential benefits of the small antagonist peptide, Motixafortide (BL-8040), targeting CXCR4 combined with Nelarabine, a nucleoside analog, is currently being tested for relapsed/refractory T-ALL (ClinicalTrials.gov: NCT02763384).

#### 3.3.3. Targeting Chemokine Receptors to Modulate Cells in the TME

The critical importance of chemokine receptors in governing immune cell migration between tissues has fostered their targeting to favor the development of a “hot” environment in cancer settings. Thus, the targeting of CCL2/CCR2 signaling to limit monocyte recruitment and M2 polarization of tumor-associated macrophages has been tested in hepatocellular carcinoma. Such strategies were based on the benefit of treatment with the RDC018 CCR2 antagonist, which induced anti-cancer CD8 T-cell responses [165], or a natural-tree-derived CCR2 antagonist, which was efficient alone and potentiated the effect of the sorafenib kinase inhibitor [166]. Along this line, Plozalizumab (MLN1202), a humanized monoclonal antibody directed against CCR2 with potential immunomodulating properties, but also antiangiogenic, antimetastatic, and antineoplastic activities, was tested in combination with an anti-CTLA4 and anti-PD1 immune checkpoint blockade in a phase II study in patients with bone metastases (ClinicalTrials.gov: NCT01015560). The effect of targeting CXCR4 on the TME is also currently being investigated in renal cell carcinoma, Waldenström macroglobulinaemia, and melanoma, upon combined treatment with the allosteric CXCR4 modulator Mavorixafor and anti-PD1 immune checkpoint blockade (ClinicalTrials.gov: NCT04274738).

### 3.4. Targeting the Atypical Receptors

Growing knowledge of ACKR expression and function paves the way toward their targeting in cancer. Because ACKR3 is highly expressed in many cancers, including oncovirus cancers, it can be used as a tumor-associated antigen for the targeted destruction of cancer cells. Furthermore, its critical role in shaping CXCL12 gradients suggests that ACKR3 inhibition could have major effects on both cancer and immune cell trafficking. Such approaches may benefit from the development of high-affinity nanobodies [167,168] or small molecules [169] targeting ACKR3. From a general perspective, the challenge in ACKR-targeted therapies lies in the broad range of chemokines they can scavenge, thus requiring further characterization of their binding specificity and function as well as the design of specific ligands that may require selective addressing to the TME.

## 4. Concluding Remarks

Although the chemokine system has led to only one approved treatment so far (i.e., CCR4 in relapsed/refractory ATLL), it carries a strong therapeutic potential for other oncovirus-associated cancers, as outlined above, but also important pitfalls related to the complex and still incompletely understood biology of the chemokine system and its role in oncogenesis. For instance, the combined targeting of several chemokines or receptors, as proceeds in some ongoing clinical trials [147], might help in achieving a more efficient clinical response, considering the interrelated functioning of the chemokine system- chemokines generally bind to several receptors and chemokine receptors often interact with several chemokines. Additionally, while chemokine-induced signaling pathways and immune-cell trafficking participate in containing latent oncovirus infection, they can also be pro-tumoral. Along this line, several chemokines have agonistic but also inverse agonistic properties on vGPCRs such as ORF74, suggesting that their modulation would have unpredictable consequences on KSHV-induced oncogenesis, depending on the stage of the viral life cycle. The direct targeting of vGPCRs, such as for HCMV-encoded US28 by monovalent nanobodies [160], could hold promises to eliminate cancer cells in combination with immunotherapies. Yet, strategies aimed at modifying the expression or function of chemokines or their receptors only at the tumor sites would improve their likelihood of clinical success. Hence, encapsulation and delivery systems have been recently reported for chemokines or siRNAs targeting chemokine receptors [170,171]. Over the last decades, a large body of work has been devoted to the design of nanodrugs and nanocarriers aimed at boosting the bioavailability of anti-cancer active substances, including their targeting and diffusion toward the multiple biological barriers that characterize tumors and their microenvironment, which still limits the success of nanodrugs in the clinic [172,173,174]. Moreover, recent advances have enabled progress in the field of 3D organoids that intend to recapitulate the structure and function of normal and neoplastic human tissue and allow the modeling of human–virus interactions, thus offering relevant systems to further delineate the impact of chemokines and receptors on oncovirus-driven disease and their potential as therapeutics in combination with cancer immunotherapy.

**Future Issues**

- What is the role of the host chemokine system in the surveillance of commensal oncovirus infection?
- What are the mechanisms that underpin the deregulation of the host chemokine system in inflamed, infected tissues and during oncogenesis in the tumor and tumor microenvironment?
- Which factors modulate the expression of the virochemokines and vGPCRs in different phases of the virus life cycle?
- How does neutralizing the expression or function of virochemokines and vGPCRs hold promise for eliminating cancer cells, and to what extent can they be selectively targeted?

## Figures and Tables

**Table 1:** Interplay between oncogenic viruses and the chemokine system.

Acronym	EBV	KSHV	HPV *	MCPyV	HBV	HCV	HTLV-1
**Full name**	Epstein–Barr virus	Kaposi’s sarcoma herpesvirus	Human papillomavirus	Merkel cell polyomavirus	Hepatitis B virus	Hepatitis C virus	Human T-lymphotropic virus 1
**Family**	*Herpesviridae*	*Herpesviridae*	*Papillomaviridae*	*Polyomaviridae*	*Hepadnaviridae*	*Flaviviridae*	*Retroviridae*
**Genome**	circular dsDNA	circular dsDNA	circular dsDNA	circular dsDNA	circular ss/dsDNA	linear +ssRNA	linear +ssRNA
**Envelop**	yes	yes	no	no	yes	yes	yes
**Genome size**	172 kb	165–170 kb	7.9 kb	5.4 kb	3.2 kb	9.6 kb	9.0 kb
**Main oncoproteins**	EBNA1, 2, 3A, 3B, 3C, LP LMP1, 2A, 2B v(Bcl-2)-BHRF1	LANA (ORF73) v-cyclin (ORF72) vFLIP (ORF71) Kaposin (K12)	E5 E6 E7	Large T Small T	HBx		Tax HBZ
**Virochemokines**		vCCL-1 (K6) vCCL-2 (K4) vCCL-3 (K4.1)					
**vGPCRs**	BILF1	ORF74					
**Main viral non coding RNA and miRNAs**	RNA EBERs 44 miRNAs from: BHRF1-miRs BARTs-miRs	25 miRNAs from: miR-K1–K9 miR-K10 miR-K12 clusters		miR-M1-3p miR-M1-5p			
**Main host chemokines targeted**		CCL1, CCL5 CXCL1-3, 5-8 CXCL4 CXCL10 CXCL12	CXCL12	CXCL8	CXCL10	CCL2 CXCL9-11	
**Main host chemokine receptors** **targeted**		CCR1-5 CCR8 CCR10 XCR1 CX3CR1 CXCR4 ACKR3	CXCR4 ACKR3				CCR4 CCR7 CCR9 CXCR4 ACKR3
**Main associated cancers**	Burkitt’s lymphoma B Cell-lymphoma NK/T Cell lymphoma Hodgkin’s lymphoma Immunodeficiency associated-lymphoproliferative disorder Nasopharyngeal carcinoma Others **	Kaposi’s sarcoma Primary effusion lymphoma Multicentric Castleman disease	Squamous cell carcinoma of uterine cervix, vulva, vagina, penis, and anus Adenocarcinoma of uterine cervix Head and neck squamous cell carcinoma (oral cavity, oropharynx, tonsil)	Merkel cell carcinoma	Hepatocellular carcinoma Cholangiocellular carcinoma	Hepatocellular carcinoma Cholangiocellular carcinoma non-Hodgkin lymphoma	Adult T-cell lymphoma/ leukemia

* “high-risk” species (alpha-5, 6, 7, 9, 11) of the mucosotropic alpha genus, and beta HPV5 and-8 type in epidermodysplasia verruciformis [6]. ** Gastric adeno-carcinoma, breast carcinoma, and EBV-associated smooth muscle tumor. dsDNA, double-stranded DNA; ssDNA, single-stranded DNA; +ssRNA, positive-sense single-stranded RNA; EBNA, Epstein–Barr Nuclear Antigen; LP, leader protein; LMP, latent membrane protein; BHRF1, Bam HI fragment H rightward open reading frame 1; LANA, latency-associated nuclear antigen; vFLIP, viral FLICE (FADD-Homologous ICE/CED-3–like) inhibitory protein; E, early; HBx, Hepatitis B virus X protein; Tax, Trans-activating transcriptional regulatory protein of HTLV-1; HBZ, HTLV-1 basic zipper factor, vGPCR, viral GPCRs.
